# Antifibrotic drug treatment of patients with idiopathic pulmonary fibrosis in Sweden: A registry-based observational study

**DOI:** 10.1177/14799731241299443

**Published:** 2024-11-12

**Authors:** Lisa Carlson, Dimitrios Kalafatis, Ida Pesonen, Jesper M Magnusson, Magnus Skold

**Affiliations:** 1 Respiratory Medicine Unit, Department of Medicine Solna, 27106 Karolinska Institutet, Stockholm, Sweden; 2 Department of Respiratory Medicine and Allergy, Karolinska University Hospital, Stockholm, Sweden; 3 Department of Respiratory Medicine, Institute of Medicine Sahlgrenska Academy, 3570 University of Gothenburg, Gothenburg, Sweden

**Keywords:** Idiopathic pulmonary fibrosis, drug therapy, body mass index, side effects, registries

## Abstract

**Objectives:**

Idiopathic pulmonary fibrosis (IPF) is characterized by progressive fibrosis of the lung parenchyma, resulting in respiratory failure. This study analysed differences in patient characteristics and antifibrotic treatment strategies during the first years after IPF diagnosis.

**Methods:**

Data from patients with IPF was extracted from the Swedish IPF registry. Patients were defined as treated (either as fully- or reduced treated) or non-treated with antifibrotic drugs. Differences in clinical parameters and side effects were defined.

**Results:**

Among 532 patients, 371 received treatment with antifibrotic drugs. Treated patients were younger, had worse lung function, higher body mass index (BMI), higher Gender-Age-Physiology stage, and were more often on oxygen treatment. Non-treated patients displayed a stable BMI, whereas patients treated with antifibrotics declined in BMI during follow-up. More than half (56%) of treated patients had reduced antifibrotic treatment. Sixty per cent reported side effects, with diarrhoea, nausea, and skin rash as the most common.

**Conclusions:**

Patients prescribed antifibrotic treatment had more advanced disease compared to patients not prescribed antifibrotics. A considerable proportion of the patients had reduced treatment, probably due to more side effects in this group. This indicates that individuals starting treatment at IPF diagnosis are considered to be in greater need of antifibrotic drug treatment by the prescriber, compared to individuals with less severe disease.

## Introduction

Idiopathic pulmonary fibrosis (IPF) is characterized by progressive fibrosis and remodeling of the lung parenchyma resulting in breathlessness, reduced work capacity, and in the end, respiratory failure and death.^1–3^ Diagnosis is based on clinical history, radiology and, in some cases, histopathology.^3^ The prevalence in Sweden is estimated at 10-40/100 000.^4^

Antifibrotic treatment with pirfenidone^5,6^ and nintedanib,^7^ slows down disease progression and is intended as a lifelong treatment. These drugs have been available in Sweden since 2012 and 2015, respectively.^8^ Both can cause side effects, mainly from the gastrointestinal and respiratory tracts, but photosensitivity reactions, headache, and fatigue are also common.^5–7^

Besides randomized controlled trials (RCTs), observational studies on the long-term use of antifibrotic drugs have contributed to a wider understanding of the effects on safety, mortality, and other outcomes.^9–15^ IPF patients treated in everyday practice differ from the well-defined cohorts of clinical trials.^16,17^ In particular, IPF patients receiving antifibrotic treatment have in observational studies been found to live longer than patients not prescribed antifibrotics.^9,13,14^ Registry based observational studies often have different baseline time points, since inclusion may not necessarily happen exactly at the time of diagnosis.^18–20^

It has been described that the treating physicians have a “wait and see” approach when an IPF patient appears to have a milder disease.^20,21^ In Sweden, all patients with IPF can receive antifibrotic drugs within the system of subsidy,^18^ and a majority of IPF patients are therefore treated, but not all.^18,19^ The reasons for not treating patients differ, but are based on individual decisions by the treating physician and the patient.^21^

Previous studies suggest that IPF patients initiating antifibrotic treatment at the time of IPF diagnosis are younger and have worse lung function compared to non-treated patients.^22,23^ Differences in healthcare systems may be one reason explaining variations in the proportions of treated patients in different international settings.^22^

Knowledge of IPF and prescription patterns in a real-world setting may help us understand and improve the management of individuals suffering from IPF. In this study, we explored baseline characteristics and longitudinal data, including survival, on treated and non-treated IPF patients within the Swedish healthcare system of subsidy. Additionally, we compared patients that were fully treated with those receiving reduced treatment.

## Methods

### The Swedish IPF-registry

The Swedish IPF-registry (SIPFR) is a quality registry including patients with a diagnosis of IPF based on national and international guideline criteria,^24–26^ and has previously been described.^19,27^ The total number of registered patients in the platform by February 2022 was approximately 800 from 24 hospitals in Sweden. All examinations and other clinical data corresponding to variables in SIPFR are carried out on an individual basis. The clinical follow-up of patients with IPF is always individual, but the general recommendations are every three to 12 months depending on disease course and the needs of the individual patient.^3,24^ Side effects are reported to SIPFR either spontaneously by the patient or after a direct question from the nurse or physician.

### Study population

Patients diagnosed with IPF between January 2014 and February 2022 with more than 3 months’ follow-up period were eligible for this study. The cohort was divided into non-treated and treated and followed over a study period of 24 months from the date of diagnosis. Patients that were not prescribed antifibrotics during the study period were defined as non-treated. Treated patients were defined as taking at least one dose of antifibrotic treatment (either pirfenidone or nintedanib) within 3 months from the date of diagnosis. Outcome was defined as either death of any cause or lung transplant during the study period. Patients who did not have an outcome were censored at the last visit during, or at the end of, the study period. Patients were defined as fully treated if they had the recommended dose^5–7^ of their antifibrotic drug and no interruptions until study end, or the final event of death, transplant, or last known follow-up. Dose reduction and treatment interruptions were allowed for up to 30 days during the study period. Treated patients not meeting these criteria were defined as having reduced treatment. Reported side effects were jointly considered in both fully and partially treated patients.

### Variables

Baseline variables were age at diagnosis, sex, gender-age-physiology (GAP)-stage,^28^ comorbidities, oxygen use, smoking status, six-minute walk test (6MWT), the ratio between forced expiratory volume in 1 s (FEV_1_) and forced vital capacity (FVC) (FEV_1_/FVC), together with percent of predicted FEV_1_ (FEV_1_%) and total lung capacity (TLC%). At baseline and follow-up, values used were body mass index (BMI), percent of predicted FVC (FVC%), and diffusion capacity for carbon monoxide (DL_CO_%). Treatment (pirfenidone and nintedanib) variables were start- and stop dates, dosing and side effects.

Baseline values collected within 3 months before or after the date of diagnosis were considered. Follow-up data were considered at 12 and 24 months (±3 months for each time point) after the date of diagnosis.

### Statistical analyses

Continuous variables were reported as mean and standard deviation. Comparisons between groups were made using t-tests and chi-squared tests when appropriate. Kaplan-Meier plots were used to illustrate survival. Survival analyses were performed using Cox proportional hazards model, adjusted for potential confounders including antifibrotic treatment, age, sex, FVC%, DL_CO_%, BMI, and supplementary oxygen treatment. The proportional hazards assumption was tested with Schoenfeld test. A two-sided *p*-value of <0.05 was considered significant. All statistical analyses were conducted in SPSS (IBM Corp. Released 2017. IBM SPSS Statistics for Windows, Version 25.0. Armonk, NY: IBM Corp) and GraphPad Prism version 9.5 (GraphPad Software, San Diego, California, USA).

### Ethical considerations

Written informed consent is required from all patients prior to inclusion in the SIPFR. The written informed consent includes the patient’s consent to publish data from SIPFR in research that has been approved by an ethical board. This study was approved by the Swedish ethical board (Dnr 2018/1449-31/1).

## Results

### Baseline characteristics

**Figure 1.**
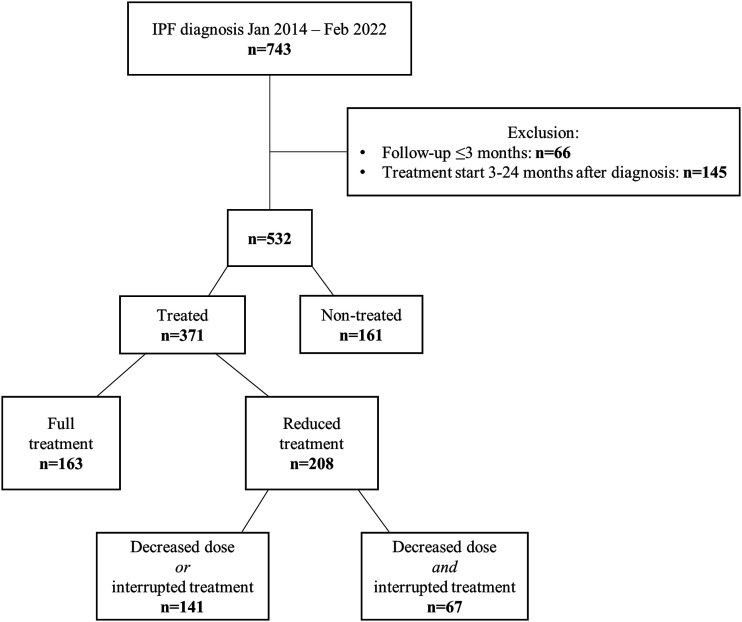
Flow chart of patient selection. *n* = number of patients.

Altogether 743 patients were eligible for this study according to the initial criteria. Exclusion criteria were met by 211 patients (Figure 1), of which 66 did not satisfy the criteria of follow-up for at least 3 months and 145 started antifibrotic treatment three to 24 months after the date of diagnosis. Baseline characteristics of these excluded patients were similar to those included, although the excluded patients had more preserved lung function in terms of FVC% and FEV1% of predicted (Table S1). A total of 532 patients were included in the study, of which 371 received antifibrotic treatment, and 161 were not treated.

Patients not treated with antifibrotics were older and had a lower BMI with less obesity (Table 1). Patients treated with antifibrotics had worse lung function with significantly lower FVC%, FEV_1_%, DL_CO_% and TLC% (*p* < .001 for all). There were, however, no differences in FEV_1_/FVC ratio, or walking distance during 6MWT. A higher proportion of non-treated patients were classified as GAP-stage 1 (52% vs 29%, *p* < .01), while a higher proportion of treated patients were classified as GAP-stage 2 (63% vs 41%, *p* < .01). Use of additional oxygen at time of the diagnosis was more common among patients that started treatment with antifibrotics (5% vs 1%, *p* < .05) (Table 1). However, the numbers of comorbidities (Table 1) did not differ between the two groups.

**Table 1.** Baseline characteristics of included patients with IPF divided into non-treated and treated.

	Non-treated (*n* = 161)	Treated (*n* = 371)	*p*-value
Sex, male (*n* (%))	120 (75)	282 (76)	0.716
Age	73.4 (7.4)	71.8 (7.4)	0.022
Ever smokers (*n* (%))	105 (75) (*n* = 141)	237 (69) (*n* = 345)	0.206
BMI	26.2 (3.4) (*n* = 94)	27.3 (4.1) (*n* = 281)	0.014
BMI <18,5 (*n* (%))	1 (1)	1 (1)	0.415
BMI 18,5-24,9 (*n* (%))	33 (35)	86 (31)	0.417
BMI 25,0-29,9 (*n* (%))	48 (51)	115 (41)	0.086
BMI >29,9 (*n* (%))	12 (13)	79 (28)	0.003
FVC%	80.62 (17.41) (*n* = 81)	70.16 (16.46) (*n* = 252)	<0.001
FEV_1_%	85.81 (17.82) (*n* = 84)	76.40 (16.98) (*n* = 262)	<0.001
FEV_1_/FVC	0.80 (0.07) (*n* = 85)	0.81 (0.08) (*n* = 253)	0.181
DL_CO_%	57.75 (18.45) (*n* = 60)	47.32 (14.01) (*n* = 219)	<0.001
TLC%	71.65 (15.66) (*n* = 55)	64.51 (12.38) (*n* = 191)	<0.001
6MWD	413 (117) (*n* = 52)	417 (123) (*n* = 195)	0.832
GAP-stage	(*n* = 56)	(*n* = 192)	0.002
1 (*n* (%))	29 (52)	56 (29)	0.004
2 (*n* (%))	23 (41)	120 (63)	0.773
3 (*n* (%))	4 (7)	16 (8)	
No. of comorbidities	1.6 (1.1) (*n* = 125)	1.5 (1.2) (*n* = 311)	0.611
Oxygen supplement (*n* (%))	1 (1)	19 (5)	0.012

Values are expressed as mean with standard deviation stated in brackets unless stated otherwise, *n* = total number, % = percent of *n*, Ever smokers = smokers and ex-smokers, BMI = body mass index, BMI <18,5 = underweight, BMI 18,5-24,9 = normal, BMI 25,0-29,9 = overweight, BMI >29,9 = obesity, FVC% = percent of predicted forced vital capacity, FEV_1_% = percent of predicted forced expiratory volume in one second, DL_CO_% = diffusion capacity of carbon monoxide, TLC% = percent of predicted total lung capacity, 6MWD = 6 min walking distance, GAP-stage = Gender, age, physiology (stage 1-3).

### Follow-up on lung function, BMI, and survival analysis

**Figure 2.**
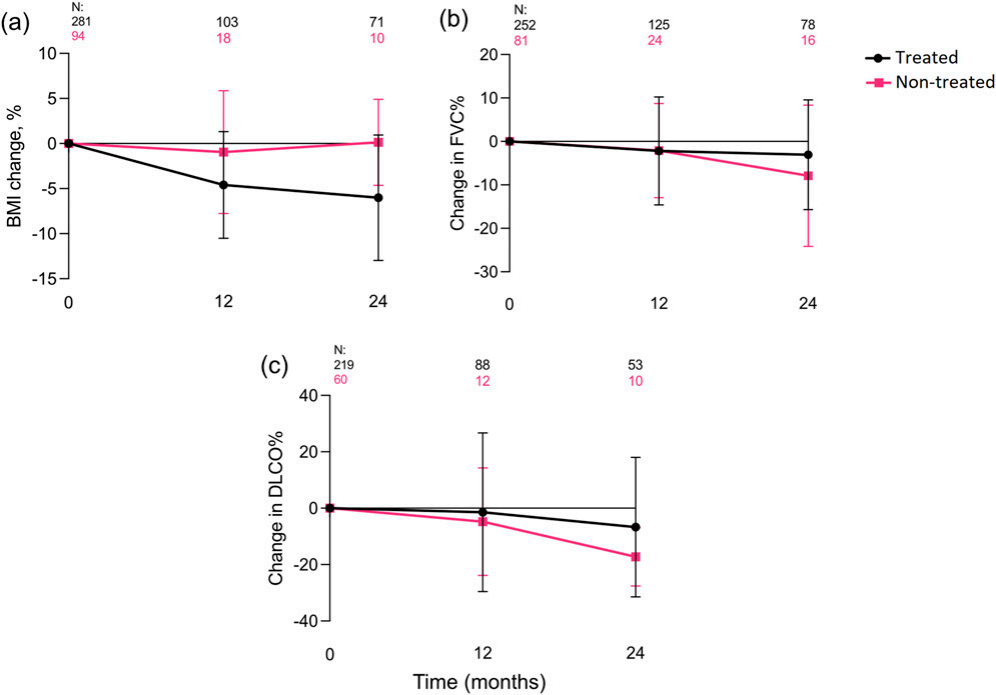
Mean (SD) change measured at baseline and follow-up visits. (a) BMI, (b) FVC%, (c) DLCO%. SD = standard deviation, *N* = number of patients, BMI = body mass index, FVC% = percent of predicted forced vital capacity, DLCO% = diffusion capacity of carbon monoxide.

Both at 12 and at 24 months, patients treated with antifibrotics declined in BMI (mean change −4,60 (SD 5,92) and −6,01 (6,96) respectively), whereas non-treated patients displayed a stable BMI (−0,94 (6,82) and 0,13 (4,78)). There was a significant difference in BMI change during follow-up between the patients treated with antifibrotics and the non-treated (Figure 2(a)). There was no difference in lung function (FVC% and DL_CO_%) during follow-up (Figure 2(b)–(c)).

**Figure 3.**
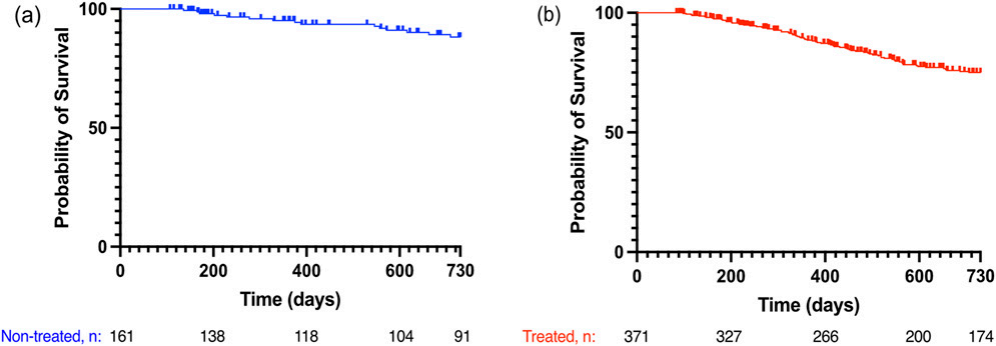
Kaplan-Meier analysis of survival of (a) non-treated patients (*n* = 161) and (b) treated patients (*n* = 371).

Since the two groups were not matched at baseline, we calculated the 2-year survival for the non-treated and treated groups separately. The 2-year survival for the non-treated patients was 91%, and for the treated Group 80% (shown in Figure 3(a)–(b)). The multivariate Cox proportional hazards model showed that higher DL_CO_% at diagnosis was associated with improved survival (HR 0.97, 95% CI: 0.94–0.99, *p* = .02) (Table 2).

**Table 2.** Multivariate Cox analysis for survival, *n* = 212.

	Hazard ratio (95% CI)	*p*-value
Antifibrotic treatment	1.11 (0.46-3.33)	0.83
Sex (female)	0.97 (0.41-2.08)	0.95
Age	0.98 (0.93-1.04)	0.47
BMI	0.94 (0.85-1.03)	0.19
DL_CO_%	0.97 (0.94-0.99)	0.02
FVC%	0.99 (0.97-1.02)	0.45
Oxygen supplementation	1.46 (0.32-4.72)	0.57

*n* = total number in the Cox analysis for survival, BMI = body mass index, DL_CO_% = diffusion capacity of carbon monoxide, FVC% = percent of predicted forced vital capacity.

### Full- or reduced treatment and side effects

Among the 371 patients in the group treated with antifibrotics, more than half (*n* = 208, 56%) had reduced treatment (Figure 1). Patients with full treatment had higher BMI (*p* < .01) and were younger (*p* < .01). Also, more women than men had reduced treatment (*p* < .01) (Table S2).

In the group of patients treated with antifibrotics, a total of 308 side effects were reported among 223 patients, while 148 patients reported none (60% vs 40%). The most common side effect was diarrhoea (reported by 48% of 223 patients), followed by nausea (21%) and skin rash (17%) (Table 3). In total, 31,3% of the fully treated patients reported side effects, compared to 82,7% of patients with reduced treatment. This difference was statistically significant (*p* < .001).

**Table 3.** Reported side effects of treated individuals.

Type of side effect	Number of patients (%), *n* = 223
Diarrhea	107 (48)
Nausea	46 (21)
Rash	37 (17)
General malaise	28 (13)
Elevated liver enzymes	25 (11)
Decreased appetite	20 (9)
Other GI-related	20 (9)
Fatigue	14 (6)
Headache and dizziness	11 (5)

% = proportion of patients reporting each side effect, *n* = total number, GI = gastrointestinal.

Furthermore, among the 208 patients with reduced treatment, 67 patients had both a reduced dose and an interrupted treatment. Altogether 100 patients had dose reduction (<300 mg nintedanib/day or <2403 mg pirfenidone/day) only, and 41 patients had interrupted treatment (pauses of >30 days and/or suspended treatment >30 days before outcome) (Figure 1). There were no differences in baseline characteristics or demographics between these three groups of patients with reduced treatment. However, patients with both dose reduction and interrupted treatment reported more side effects compared to patients with either reduced dose or interrupted treatment (96% vs 77%, *p*-value <.001).

## Discussion

We demonstrate that a considerable proportion of patients with IPF in Sweden do not receive antifibrotic treatment, although it is possible for all IPF patients within the healthcare system to receive subsidy. These patients differed in many aspects from those receiving antifibrotic treatment. Compared to treated patients, the non-treated patients were older, less obese, had better lung function, and lower GAP stage at baseline. The non-treated patients had a 2-year survival of 91% with stable BMI. In contrast, the treated patients, with more advanced disease at baseline, had a 2-year survival of 80% and a decline in BMI. Due to the differences in baseline health status, any observed outcomes could not be reliably attributed to the effects of treatment alone. As a result, no formal comparison of outcomes between the two groups was conducted in this analysis. Thus, the baseline, follow-up, and survival differences between the treated and the non-treated groups in this study indicate that the non-treated group is, already at diagnosis, a group of patients with milder disease. We also demonstrate that more than half of the patients had reduced antifibrotic treatment and that these patients were younger, more often women and had lower BMI at treatment start, compared to patients with full treatment.

All-cause mortality in the non-treated group was 9% 24 months after diagnosis. In pooled data from clinical trials,^29^ the mortality rate was 14% 2 years after baseline in the placebo groups. In our study, patients had higher FVC% and DL_CO_% than the pooled trial data. Our patients also used less additional oxygen, suggesting a less severe disease. In contrast to the inclusion criteria applied in the clinical trials,^5–7^ our study explores other contexts where antifibrotic treatment is initiated; inclusion in our study was at the date of IPF diagnosis and treatment initiated within 3 months, and there were no criteria for FVC%, DL_CO_% or distance at 6MWT. Thus, the non-treated patients in our study were not comparable to the placebo groups in clinical trials.

During the observation period, BMI declined among the treated patients while it was stable in patients not treated with antifibrotics. Previous studies suggest that BMI decline is a prognostic marker, with a greater decrease over 1 year being associated with an increased risk of death or lung transplant in the subsequent 12 months.^30–35^ Interestingly, a previous study reported that BMI decline can predict mortality even in patients without a decline in FVC.^35^ The reason for BMI decline was not addressed in this study. However, disease severity and side effects of antifibrotic treatment, or a combination of both these factors, may be plausible explanations. Our results emphasise the importance of monitoring weight and weight loss among patients with IPF, and especially among patients with severe disease and in those receiving antifibrotic treatment. The cause for the difference in decline in BMI between treated and non-treated patients should be further explored. Additional studies are also warranted by the small numbers of patients included in our longitudinal analysis of BMI and lung function.

The difference in survival between the non-treated and the treated group in our study is most likely an effect of the differences in disease severity at baseline. The multivariate regression model indicated that DL_CO_% is an important factor that influences mortality while treatment with antifibrotics has no impact on mortality. Our results suggest that, in a clinical context, a less severe disease at diagnosis might lead to reluctance by the treating physician to prescribe antifibrotic treatment. Physicians’ reasons not to initiate antifibrotic treatment are previously described as patients having stable disease, preserved lung function and being older.^21^ The worse survival in the group prescribed antifibrotics supports the hypothesis that the individuals starting treatment close to IPF diagnosis do not do this by chance but are considered to be in greater need of antifibrotic drug treatment than individuals with less severe disease.^22^

The type of side effects in our study are similar to those described in clinical trials^5–7^ and observational studies.^12,14,15^ Our study demonstrates that more than half of the patients have reduced treatment and report multiple side effects. Further, patients with fewer reported side effects more often have full dose with no interruptions. Porse et al.^14^ have reported that reducing the dose of the antifibrotic agent result in only a minor difference in survival between full dose treated and reduced dose treated. Among patients with reduced treatment in our study, one third had both reduced dose and interrupted treatment. This group reported more side effects than patients with only dose reduction or treatment interruptions. This is coherent to guidelines for managing side effects of antifibrotic drugs,^24,36^ where a higher number of side effects would result in an increase in treatment adjustments (i.e., decreased dose and treatment pauses). Hence, we interpret our results as having adverse effects result in prescription of a lower dose, and not that using a reduced dose is associated with more side effects.

This observational study used registry data, making it possible to use an extensive data set with a follow-up period, starting at diagnosis as the baseline, including both treated and non-treated patients. By contrast, in RCTs,^5–7^ the subjects may have had IPF diagnosis up to 5 years earlier. When using registry data, the diagnoses are not confirmed by central monitoring, as in the RCTs, but multidisciplinary discussion is recommended when diagnosing IPF in clinical practice.^3,17,24^ One of the strengths of using registry data is the inclusion of a more heterogeneous patient population than in RCTs, covering more of the population seen in clinical practice. Furthermore, using the time of diagnosis as the baseline in this study not only results in a standardized baseline for all patients, but also describes the first years of the disease course for both treated and non-treated patients.

A better survival is previously reported^9,14^ together with less decline in FVC%^13^ in patients treated with antifibrotics compared to those not treated. Previous studies have used various methods, and baseline values are not always considered at the same event in the treated patients as in the non-treated patients, risking immortal time bias and overestimation of treatment effects.^37^ Another variation between observational studies is when to consider a patient as treated, with criteria ranging from the intention-to-treat approach to the criteria of antifibrotic use for at least 6 months.^9,12,14,15^ This study contributes with a well-defined baseline, i.e. the date of IPF diagnosis, and therefore, the results display the heterogeneity of the IPF patients.

Variables collected in SIPFR are used in this observational study. Hence, other residual factors may be possible confounders, in particular in our longitudinal follow-up. For example, it is known that pulmonary rehabilitation has beneficial effects on 6MWD, dyspnea, and HRQL, although the effect on survival is uncertain. The effect of nutritional support may also be a plausible confounder.^38^

The missing data in this study is a limitation, and results related specifically to the longitudinal findings should be interpreted cautiously and considered hypothesis-generating. In the non-treated group, the proportion of missing GAP-stage values was significantly higher compared to the treated group due to a larger amount of missing FVC% and/or DL_CO_% values. This may reflect the milder disease that the non-treated group presents with since monitoring patients with milder disease is less frequent than patients with more advanced disease.^21,24–26^ Hence, the missing data in our study should not be considered a protocol deviation, as they are part of the clinical routine and management of the disease. Furthermore, data from the Swedish prescription registry could have been added to validate that the patients actually used the drugs. However, using SIPFR-data was considered sufficient in this study since initiated antifibrotic treatments are clinically monitored,^8,24^ as are the entered treatment data in SIPFR.

In this study we tested if the differences previously observed in other registry based observational studies,^22^ also could apply to a different healthcare setting, as the one used in Sweden. The reimbursement systems differ between various countries, as Pesonen et al^18^ previously described. The data obtained from national registries are important sources since it is known that these IPF patients differ from those that were included in the RCTs, and results from registry based observational studies should be considered in the context of the specific nations’ healthcare system.^18^ Similar in the US and in Sweden, although very different health care systems, IPF patients starting treatment are younger with worse lung function compared to individuals not initiating treatment. These results indicate that the prescribing physician generally takes the individual IPF patients’ characteristics into consideration.^21^ Recommendations of early initiation of antifibrotic treatment to all patients with IPF was introduced during the data collection of this study.^26^

## Conclusions

Patients starting antifibrotic treatment at the time of diagnosis have more advanced disease compared to those who are not prescribed this treatment. More than half of the patients on antifibrotics receive reduced treatment, with more side effects occurring in the group of patients with more treatment adjustments, highlighting the importance of clinical management of side effects. There are also differences in age, BMI, and sex between patients with full and reduced treatment. These differences need to be further explored with emphasis on possible associations. Our results demonstrate disparities in the prescription of antifibrotic treatment, treatment patterns, and occurrence of side effects in a Swedish context. This study also put emphasis on the importance of considering heterogeneity among IPF patients both in research and in clinical work.

## Supplemental Material

Supplemental Material - Antifibrotic drug treatment of patients with idiopathic pulmonary fibrosis in Sweden: A registry-based observational study Supplemental Material for Antifibrotic drug treatment of patients with idiopathic pulmonary fibrosis in Sweden: A registry-based observational study by Lisa Carlson, Dimitrios Kalafatis, Ida Pesonen, Jesper M Magnusson and Magnus Skold in Chronic Respiratory Disease.

## Data Availability

The data that support the findings of this study are not publicly available due to containing pseudonymized information and applying to the general data protection regulation (GDPR), but are available from the corresponding author (LC) upon reasonable request.
