# Supplementary material for: Antifibrotic drug treatment of patients with idiopathic pulmonary fibrosis in Sweden: A registry-based observational study
Source: Chron Respir Dis. 2024 Nov 12;21:14799731241299443. doi: 10.1177/14799731241299443 (PMC11558727; doi:10.1177/14799731241299443)
Supplement: Supplemental Material - Antifibrotic drug treatment of patients with idiopathic pulmonary fibrosis in Sweden: A registry-based observational study [file sj-pdf-1-crd-10.1177_14799731241299443.pdf]

Table S1. Baseline characteristics of excluded patients, initiating antifibrotic treatment 3-24 months from diagnosis, and included patients initiating treatment within 3 months from diagnosis.

	Excluded patients, n=145	Included patients, n=532	p-value
**Sex, male (n (%))**	101 (70)	402 (76)	0.149
**Age**	72.7 (6.4)	72.3 (7.4)	0.477
**Ever smokers (n (%))**	99 (72) (n=137)	342 (70) (n=486)	0.667
**BMI**	27.2 (4.4) (n=135)	27.1 (3.9) (n=375)	0.789
**FVC%**	79.66 (16.12) (n=61)	72.70 (17.27) (n=333)	0.004
**FEV_1%**	83.81 (16.72) (n=63)	78.69 (17.63) (n=346)	0.033
**FEV_1/FVC**	0.80 (0.08) (n=61)	0.81 (0.08) (n=338)	0.314
**DL_{CO}%**	51.38 (12.98) (n=61)	49.56 (15.64) (n=279)	0.398
**TLC%**	69.33 (11.63) (n=45)	66.11 (13.48) (n=246)	0.134
**6MWD**	421 (116) (n=44)	417 (122) (n=247)	0.804

Values are expressed as mean with standard deviation stated in brackets unless stated otherwise, n=total number, %=percent of n, Ever smokers=smokers and ex-smokers, BMI=body mass index, FVC%=percent of predicted forced vital capacity, FEV_1%=percent of predicted forced expiratory volume in one second, DL_{CO}%=diffusion capacity of carbon monoxide, TLC%=percent of predicted total lung capacity, 6MWD= 6 minute walking distance

Table S2. Baseline characteristics and demographics on patients with full and reduced treatment.

	Full treatment (n=163)	Reduced treatment (n=208)	p-value
**Sex, male (n (%))**	138 (85)	144 (69)	0.001
**Age**	70.4 (7.4)	72.9 (7.3)	0.001
**Ever smokers (n (%))**	112 (74) (n=151)	125 (65) (n=194)	0.053
**BMI**	28.1 (3.4) (n=121)	26.8 (4.0) (n=160)	0.005
**BMI <18,5 (n (%))**	0 (0)	1 (1)	0.384
**BMI 18,5-24,9 (n (%))**	29 (24)	57 (36)	0.036
**BMI 25,0-29,9 (n (%))**	50 (41)	65 (41)	0.906
**BMI >29,9 (n (%))**	42 (35)	37 (23)	0.032
**FVC%**	68.41 (14.97) (n=114)	71.60 (17.51) (n=138)	0.126
**FEV_1%**	76.68 (17.75) (n=115)	76.19 (16.42) (n=147)	0.818
**FEV_1/FVC**	0.82 (0.08) (n=115)	0.81 (0.09) (n=138)	0.476
**DL_{CO}%**	47.10 (14.18) (n=99)	47.49 (13.93) (n=120)	0.838
**TLC%**	64.06 (12.92) (n=86)	64.89 (11.96) (n=105)	0.647
**6MWD**	426 (122) (n=84)	411 (124) (n=111)	0.392
**GAP-stage**	(n=91)	(n=103)	
**1 (n (%))**	24 (26)	33 (32)	0.387
**2 (n (%))**	57 (63)	64 (62)	0.943
**3 (n (%))**	10 (11)	6 (6)	0.192
**No. of comorbidities**	1.5 (1.2) (n=138)	1.6 (1.2) (n=173)	0.440
**Oxygen supplement (n (%))**	9 (6)	10 (5)	0.757

Values are expressed as mean with standard deviation stated in brackets unless stated otherwise, n=total number, %=percent of n, Ever smokers=smokers and ex-smokers, BMI=body mass index, BMI <18,5=underweight, BMI 18,5-24,9=normal, BMI 25,0-29,9=overweight, BMI >29,9=obesity, FVC%=percent of predicted forced vital capacity, FEV_1%=percent of predicted forced expiratory volume in one second, DL_{CO}%=diffusion capacity of carbon monoxide, TLC%=percent of predicted total lung capacity, 6MWD= 6 minute walking distance, GAP-stage= Gender, age, physiology (stage 1-3)
